# TET1-mediated DNA demethylation suppresses migration of goat placental trophoblast cells

**DOI:** 10.1093/jas/skaf378

**Published:** 2025-10-30

**Authors:** Zhipeng Sun, Yiping Tang, Yuhao Liao, Xiang Ma, Mingming Wang, Xingqiang Fang, Songjian Yang, Junyin Zhao, Yongju Zhao

**Affiliations:** College of Animal Science and Technology, Southwest University, Chongqing, 400715, China; Chongqing Key Laboratory of Herbivore Science, Chongqing, 400715, China; Chongqing Engineering Research Center for Herbivores Resource Protection and Utilization, Chongqing, 400715, China; Animal Nutrition Institute, Sichuan Agricultural University (SAU), Chengdu, 625014, China; College of Animal Science and Technology, Southwest University, Chongqing, 400715, China; College of Animal Science and Technology, Southwest University, Chongqing, 400715, China; College of Animal Science and Technology, Southwest University, Chongqing, 400715, China; College of Animal Science and Technology, Southwest University, Chongqing, 400715, China; College of Animal Science and Technology, Southwest University, Chongqing, 400715, China; College of Animal Science and Technology, Southwest University, Chongqing, 400715, China; College of Animal Science and Technology, Southwest University, Chongqing, 400715, China; Chongqing Key Laboratory of Herbivore Science, Chongqing, 400715, China; Chongqing Engineering Research Center for Herbivores Resource Protection and Utilization, Chongqing, 400715, China

**Keywords:** cell migration, epigenetic regulation, goat, placental trophoblast cells, TET1

## Abstract

The placenta is essential for supporting fetal growth by delivering nutrients and removing waste products. Its development hinges on precise epigenetic regulation, yet the role of Ten-eleven translocation 1 (TET1) in ruminant trophoblast biology remains unclear. Here, we show that TET1 acts as a suppressor of goat placental trophoblast cells (GTCs) migration. Using immortalized GTCs, we induced TET1 overexpression via 20 ng/μL doxycycline (Dox) and achieved knockdown through shRNA (piLenti-shTET1-GFP-a). Overexpression of TET1 markedly reduced GTCs migration and viability, whereas knockdown enhanced migration, with these effects reversible by restoring TET1 expression using Dox. Mechanistically, TET1 influenced chemical changes to DNA that control gene activity, decreasing DNA methylation mark (5mC) while increasing hydroxymethylation mark (5hmC). Conversely, TET1 knockdown had the opposite effect on these marks. Our results establish TET1 as a key suppressor of goat trophoblast cell migration via DNA modifications, providing novel insights into placental development and potential strategies for improving reproductive efficiency in livestock.

## Teaser

This study identifies TET1 as a key epigenetic regulator of goat trophoblast cells, suppressing cell migration through modulation of DNA methylation. Our findings provide novel molecular insights into placental development and offer potential strategies to improve reproductive efficiency in ruminants.

## Introduction

The placenta is a critical organ that mediates the exchange of nutrient, gases, and signals between the mother and fetus during pregnancy. Its proper structural and functional development is indispensable for embryo implantation, fetal growth, and the maintenance of gestation (Burton and Fowden, 2015). Despite its importance, reproductive efficiency remains a major challenge in mammals, with fetal loss during early pregnancy reported at ≥ 30% in domestic animals and > 50% in humans (Grazul-Bilska et al., 2011). Trophoblast cells (TCs), the predominant cellular component of the placenta, not only contribute to placental structure but also play essential roles in implantation through migration, invasion, and differentiation (Zang et al., 2024). In ruminants such as sheep and goats, trophoblast cell function directly determines placental development and fetal health ­(Davenport et al., 2023; Jia et al., 2023). Emerging as the first differentiated lineage during the morula stage, trophoblast cells are indispensable for placental morphogenesis and maternal–fetal interactions (Knöfler and Pollheimer, 2013). Their invasive and migratory capacities are prerequisites for successful placentation, embryonic development, and favorable pregnancy outcomes (Arutyunyan et al., 2023; Zang et al., 2024). Recent studies highlight the crucial role of epigenetic regulation, particularly DNA hydroxymethylation mediated by Ten-eleven translocation (TET) enzymes, in trophoblast differentiation, placental development, and maternal–fetal crosstalk. TET-mediated DNA demethylation establishes the epigenetic landscape necessary for placental lineage specification, thereby exerting profound effects on pregnancy outcomes (Vasconcelos et al., 2023). Thus, understanding the molecular mechanisms by which TET1 regulates trophoblast migration is of great significance for improving reproductive efficiency in goats and other ruminants.

In the mammalian reproductive system, TET1 has been implicated in embryonic development, placentation, and the maintenance of stem cell pluripotency (Yamaguchi et al., 2012). For example, genome-wide DNA demethylation in primordial germ cells (PGCs) occurs dynamically at three development stages (E8.5, E10.5, and E10.5–E13.5) through changes in 5mC and 5hmC levels (Yamaguchi et al., 2012; Yamaguchi et al., 2013; Hargan-Calvopina et al., 2016). TET-dependent demethylation has been shown to regulate multiple reproductive processes, including oocyte maturation, embryo implantation, placentation, pregnancy maintenance, and offspring programming (Chen et al., 2023; Huang et al., 2024; Lu et al., 2024; Montgomery et al., 2024; Zhang et al., 2024b). The TET enzyme family comprises three homologs–TET1, TET2, and TET3–each exhibiting distinct spatiotemporal expression patterns during early embryogenesis: TET1 is highly expressed during the morula stage, TET2 from the zygote to blastocyst, and TET3 primarily at the zygote stage (Ma et al., 2014; Zhang et al., 2023). Notably, TET1 plays a pivotal role in the post-implantation stage by regulating gene expression programs critical for embryonic development (Khoueiry et al., 2017; Stötzel et al., 2024). As a central family member, TET1 is involved in gene regulation, cell fate determination, and developmental processes across various cell types (Wu and Zhang, 2017; Jabbari et al., 2025). Previous studies have shown that TET1 modulates trophoblast proliferation and differentiation through methylation changes in key developmental genes (Jin et al., 2014), while TET2 activates genes such as ENPEP to promote trophoblast differentiation and implantation potential (Huang et al., 2024). Despite these insights, the precise role and molecular mechanisms of TET1 in regulating trophoblast migration and DNA methylation in goat placental trophoblast cells remain poorly defined. Despite extensive studies in human and cancer models, the precise role of TET1 in regulating trophoblast migration in ruminants remains largely unexplored. Addressing this gap is crucial for understanding ruminant-specific placental biology.;

Therefore, the present study aimed to investigate the function of TET1 in GTCs, with a particular focus on its role in regulating cell migration and DNA methylation status. Using a doxycyline-inducible TET1 overexpression system and shRNA-mediated knockdown in immortalized GTCs, combined with functional assays, we sought to elucidate the contribution of TET1 to trophoblast biology. This work provides new insights into the epigenetic regulation of goat placental development and may offer a theoretical basis for improving reproductive performance and addressing fertility challenges in ruminants.

## Materials and Methods

### Ethical statement

This study did not involve the use of live animals. Immortalized goat trophoblast cells (GTCs) were established from placental tissue collected at Tengda Animal Husbandry Co., Ltd, Da’zu, Chongqing, China. The collection and use of the original tissue, as well as subsequent cell line establishment, were approved by the Animal Welfare and Ethics Committee of Southwest University (Chongqing, China) (Approval No. IACUC-20170706-045). All experimental procedures complied with institutional and national guidelines for the care and use of animals.

### Culture of goat trophoblast cells (GTCs)

Immortalized goat trophoblast cells (GTCs) were originally derived from placental tissue collected and subsequently immortalized. The cell line was authenticated by immunofluorescence staining of trophoblast-specific markers including cytokeratin-7 (CK7) and placental lactogen, as previously described (Petroff et al., 2006). Cells were maintained in Dulbecco’s modified Eagle’s medium (DMEM; Gibco, USA; No. 11965092) supplemented with 10% fetal bovine serum (FBS; Gibco, USA; No. 10099141) and 1% penicillin–streptomycin (100 U/mL penicillin, 100 μg/mL streptomycin; Gibco, USA; No. 15140122). Cells were incubated at 37 °C in a humidified atmosphere containing 5% CO_2_. For routine passage, cells at 70–80% confluence were washed twice with phosphate-buffered saline (PBS; Gibco, USA; No. 70013032) and digested with 0.25% trypsin–EDTA (Gibco, USA; No. 25200056) for 2–3 min at 37 °C. The reaction was terminated with complete medium, and cells were collected by centrifugation (1,000 rpm, 5 min). The cell pellet was resuspended in fresh complete medium and seeded into new culture flasks at a ratio of 1:3. For long-term storage, cells in logarithmic growth phase were digested with 0.25% trypsin, centrifuged, and resuspended in 1 mL freezing medium DMEM/F-12: FBS: DMSO = 6:3:1 (DMSO; Sigma-Aldrich, USA; No. D2650) per cryovial. The suspension was aliquoted into cryovials and gradually cooled to −80°C before being transferred to liquid nitrogen for long-term storage. For recovery, frozen cells were rapidly thawed in a 37 °C water bath, transferred to complete medium, centrifuged to remove DMSO, and reseeded for culture. Cell viability was assessed using Trypan Blue exclusion, and cell density was determined with a hemocytometer. Only cultures with a viability of >90% were used for experiments. Cell viability was calculated as:


Cell viability (%) = (Number of viable cells/Total number of cells) × 100%

### Doxycycline (Dox) treatment of GTCs

Logarithmically growing GTCs were seeded into T25 flasks to ensure uniform growth. After overnight stabilization in complete medium, cells were cultured in fresh complete medium containing 0 ng/µL (control), 1 ng/µL, 10 ng/µL, or 20 ng/µL Dox (Sigma-Aldrich, USA; No. D9891) for 72 h (*n* = 3 per group, four groups in total), followed by collection for downstream experiments. Based on previous studies and our preliminary tests showing effective induction without significant cytotoxicity, 20 ng/µL Dox was chosen as the highest concentration for subsequent experiments.

### shRNA transfection

Four shRNA constructs targeting different regions of the goat TET1 gene (GenBank: KU870424.1) were designated (piLenti-shTET1-GFP-a, -b, -c, and -d; sequences listed in Table 1) and synthesized. Among these, construct ‘a’ exhibited the highest knockdown efficiency and was used for subsequent experiments. Plasmids containing shRNA constructs targeting goat TET1 were generated and propagated in *E. coli*, then amplified in LB broth with kanamycin at 37 °C, 150 rpm. Plasmids were purified using the E.Z.N.A. Endo-free Plasmid DNA Mini Kit II (Omega Bio-Tek, USA; No. D6942-02) and verified by sequencing and restriction digestion. Goat trophoblast cells (GTCs) were seeded in 6-well plates at 80% confluence and transfected with 4 µg plasmid DNA using DNAfectin Plus (GenScript, China; No. AT0003) in 200 µL serum-free, antibiotic-free medium, incubated at room temperature for 20 min, and added to cells for 12–16 h. The medium was then replaced with complete medium, and cells were harvested after 48 h. Transfection efficiency was evaluated by fluorescence microscopy, and knockdown efficiency was validated by RT-qPCR and immunofluorescence staining.

**Table 1.** Interference sequence of TET1 gene

shRNA name	Sequence
**Pilenti-shTET1-GFP-a**	5′-GTCATCAAACTCAGAGATAATTTTGGTAC-3′
**Pilenti-shTET1-GFP-b**	5′-CCAGATCCTGTGAACTGCAACTCAAATAG-3′
**Pilenti-shTET1-GFP-c**	5′-ACAGTGAATGTTAATCAGAAAGCTCATCC-3′
**Pilenti-shTET1-GFP-d**	5′-TCATTTGGTGTTATTCCTCAAGATGAGCA-3′

### RNA extraction

Total RNA was extracted using TRIzol reagent (Invitrogen, USA; No. 15596026) according to standard protocols, including cell lysis, chloroform extraction, isopropanol precipitation, and 75% ethanol wash. RNA was dissolved in DEPC-treated water, and concentration and purity were measured spectrophotometrically (OD260/OD280 = 1.8–2.0). Integrity of 28S and 18S rRNA was assessed by agarose gel electrophoresis. cDNA synthesis was performed using the TIANGEN FastQuant RT Kit (with gDNA) (TIANGEN, China; No. KR106), with the reaction mixture containing 2 µL 10× Fast RT Buffer, 1 µL RT Enzyme Mix, 2 µL FQ-RT Primer Mix, and 5 µL RNase-free ddH_2_O. Reverse transcription was conducted at 42 °C for 15 min, followed by 95 °C for 3 min.

### RT-qPCR

Reverse transcription-quantitative polymerase chain reaction (RT-qPCR) was performed using 50 ng/µL cDNA template in a 10 µL reaction containing 0.5 µL each primer and 5 µL 2× GoTaq qPCR Master Mix (Promega, USA; No. A6001). Thermal cycling was 95 °C for 10 min, then 39 cycles of 95 °C for 15 s and Tm annealing for 1 min. Ct values were generated automatically. Goat β-actin served as the internal reference. Primers for TET1, TET2, TET3, and β-actin were designed using Primer 5.0 (Table 2).

**Table 2.** Primers list

Gene symbol	Primers sequence (5′→3′)	Products length
***TET1***	F: CATCACTGTCCGTCTTTGGA	126
	R: ACTTTCCTGTGCCTTTGTGG	
***TET2***	F: GATACCATCTCCGTCTCCCATT	109
	R: CTTGCTGTCTCCATTCACTTCC	
***TET3***	F: CCTTCTCCTTCGGTTGTTCCTG	113
	R: CACTTCTTCCTCTTTGGGGTTGTC	
	R: CACTTGGCACACCATCATCT	
**β-actin**	F: GAACCCCAAGGCCAACCG	145
	R: CCCGTCCCCAGAGTCCAT	

### Immunocytochemistry (ICC/immunofluorescence staining)

For immunofluorescence staining (here referred to as ICC), GTCs were seeded on pretreated coverslips in 24-well plates. At ∼80% confluence, cells were washed with PBS, fixed with 4% paraformaldehyde (Sigma-Aldrich, USA; No. 158127) for 30 min, permeabilized with 0.5% Triton X-100 (Sigma-Aldrich, USA; No. T8787) for 20 min, and blocked with 10% goat serum (Boster, China; No. AR1009) for 60 min. Cells were then incubated with primary antibodies of Anit-TET1 (Abcam, UK; No. ab191698), Anit-5mC (Active Motif, USA; No. 39649), and Anit-5hmC (Active Motif, USA; No. 39769) at 4 °C for 16 h, followed by fluorescently labeled secondary antibodies (goat anti-rabbit IgG Alexa Fluor 488; Thermo Fisher Scientific, USA; No. A-11034) at room temperature in the dark for 2 h. Nuclei were counterstained with DAPI (Sigma-Aldrich, USA; Cat. No. D9542) or Hoechst (Thermo Fisher Scientific, USA; No. H3570), and samples were mounted with anti-fade medium before observation under a fluorescence microscope.

### Cell scratch assay

Parallel reference lines (0.5–1 cm apart) were drawn on the back of 24-well plates. Log-phase cells were seeded at 1 × 10^5^/mL (0.5 mL per well) and cultured overnight to form a confluent monolayer. A 200 µL sterile pipette tip was used to create linear scratches along the reference lines. Cells were washed thrice with PBS to remove debris and cultured in respective treatment media. Images were captured at 0, 1, 2, and 3 days to monitor wound closure.

### Cell viability assay (MTS)

Cell viability was determined using the MTS Assay Kit (Promega, USA; No. G3580). The MTS tetrazolium compound 3-(4,5-dimethylthiazol-2-yl)-5-(3-carboxymethoxyphenyl)-2-(4-sulfophenyl)-2H-tetrazolium is bioreduced by metabolically active cells into a colored formazan product. Cells in log-phase were seeded into 96-well plates at 1 × 10^5^/mL (200 µL/well) and cultured overnight. Medium was replaced with treatment-specific medium. At 0, 1, 2, and 3 days, 20 µL MTS reagent and 100 µL serum-free DMEM/F12 were added per well, incubated in the dark for 4 h, and absorbance measured at 490 nm. Cell viability was calculated as:


$$Relative\;cell\;viability\;\left(\%\right)\;=\;\left(OD_{experiment}-OD_{blank}\right)/\left(OD_{control}-OD_{blank}\right)\;\times \;100\%$$

### Statistical analysis

Relative gene expression was calculated using the 2^−ΔΔCt^ method (ΔΔCt = Ct_target-mean ΔCt_reference). All experiments were performed in triplicate. Statistical analysis and graphing were performed using Prism 10.0 software (GraphPad Software, USA). RT-qPCR data were analyzed by independent-sample t-test, and results are presented as mean ± SD. Differences were considered statistically significant at **P < 0.01 and *P < 0.05.

## Results

### Dox-induced overexpression of TET1 in GTCs

**Figure 1.**
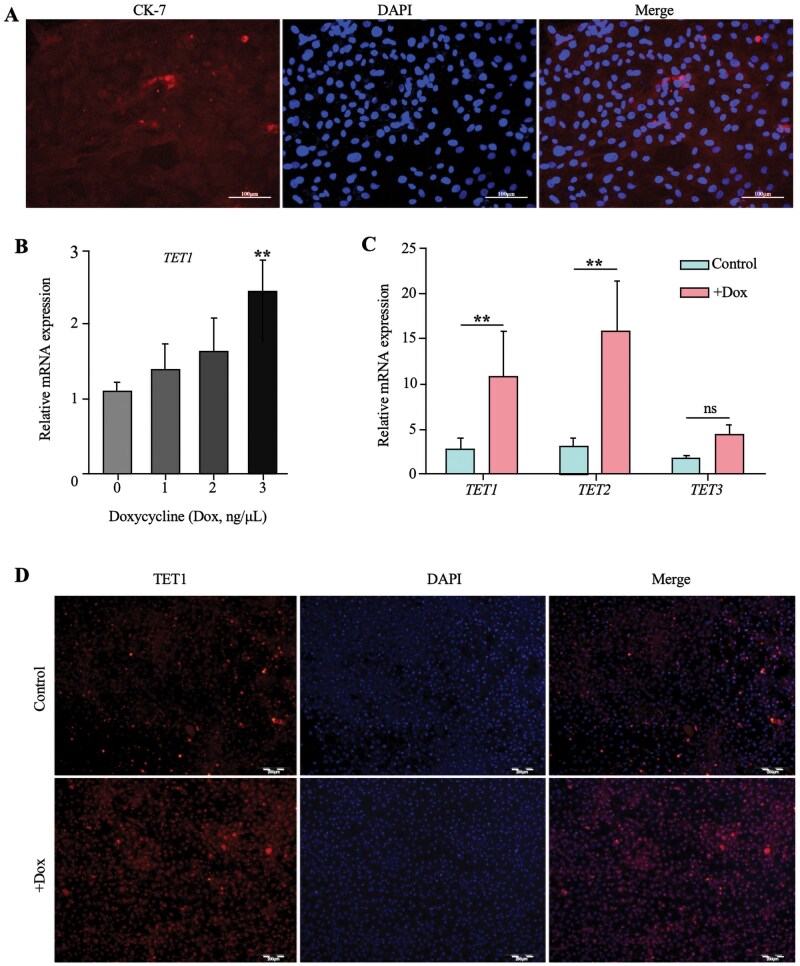
Cell identification and Dox-induced upregulation of TET1 expression in GTCs. (A) Immunocytochemical identification of GTCs, scale bar = 100 μm. (B) RT-qPCR analysis of *TET1* mRNA level in GTCs treated with different concentrations of Dox treatment. (C) RT-qPCR analysis of *TET* family mRNA levels in GTCs treated with 20ng/μL concentrations of Dox. (D) ICC staining analysis of TET1 protein level in GTCs with 20ng/μL Dox treatment. Scale bar = 200 μm. **P < 0.01, ns *P* > 0.05.

Cytokeratin-7 (CK-7) is a well-established marker protein of placental chorionic trophoblast cells (Zhang et al., 2016). In our study, ICC demonstrated that cultured GTCs exhibited weak but positive expression of CK-7, as indicated by Cy3 red fluorescence (Figure 1C), thereby confirming their trophoblastic identity. Doxycycline (Dox) activates the Tet-On system by fusing the bacterial Tet repressor (TetR) with a eukaryotic transcriptional activation domain (e.g., VP16), forming rtTA that induces TET1 transcription. Previous studies have shown that Dox-induced TET1 overexpression suppresses cancer cell proliferation and tumor growth (Neri et al., 2015). Here, we applied this system to induce TET1 overexpression in GTCs and assessed its functional effects.

To determine the optimal concentration of Dox, four doses (0, 1, 10, and 20 ng/µL) were tested based on prior reports (Zheng et al., 2016; Tomioka et al., 2017). RT-qPCR analysis demonstrated dose-dependent induction of TET1 expression (Figure 1B). At 20 ng/µL, TET1 mRNA levels were significantly higher than in the other groups (*P *< 0.01). Consequently, 20 ng/µL was selected as the optimal concentration. Further analysis showed that 20 ng/µL Dox increased TET1 and TET2 mRNA expression (*P *< 0.01), while TET3 expression remained unchanged (*P *> 0.05) (Figure 1C). ICC confirmed nuclear localization of TET1 protein and a marked increase in expression in the 20 ng/µL group compared with controls (Figure 1D), consistent with mRNA results. These findings establish 20 ng/µL Dox as an effective condition for TET1 induction and it was used in all subsequent experiments.

### Dox-mediated TET1 overexpression suppresses GTC viability and migration

**Figure 2.**
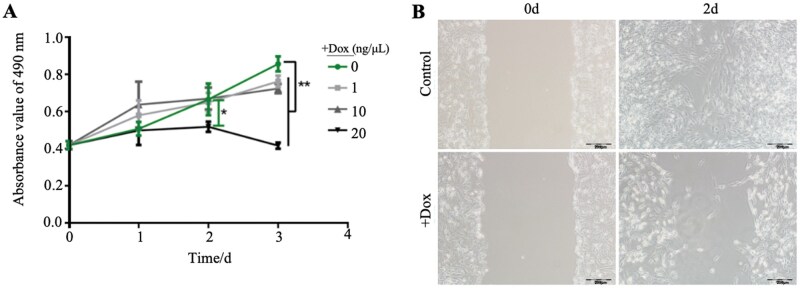
Dox promotes TET1 expression, suppressing GTC viability and migration capacity. (A) MTS analysis of activity in GTCs treated with different concentrations of Dox. (B) Scratch test analysis of migration ability in GTCs with 20ng/μL Dox treatment. Scale bar = 200 μm. *P < 0.05, **P < 0.01.

To investigate the effects of TET1 overexpression, GTCs were treated with Dox and assessed for viability and migration. MTS assays revealed that Dox affected cell viability in a concentration- and time-dependent manner (Figure 2A). On day 1, low-dose groups (1 and 10 ng/µL) showed slightly higher viability than controls (0 ng/µL), while 20 ng/µL produced a pronounced inhibitory effect. By day 2, viability in the 20 ng/µL group decreased significantly (22.13%, *P *< 0.05), with no significant changes in other groups (*P *> 0.05). By day 3, viability decreased by 10.98%, 15.49%, and 51.42% in the 1, 10, and 20 ng/µL groups, respectively (*P *< 0.01). These results indicate that 20 ng/µL Dox treatment for 2 days is optimal for inducing TET1 expression with measurable functional effects. Given the tumor-like migratory behavior of trophoblast cells (Carvajal et al., 2021), wound-healing assay were used to assess the impact of TET1 overexpression. After 2 days of treatment, migration distance decreased from 490.75 µm in controls to 177.17 µm, a 63.90% reduction (Figure 2B). Thus, TET1 overexpression significantly inhibited GTCs migratory as well as viability.

### Dox-mediated TET1 overexpression reduces DNA methylation in GTCs

**Figure 3.**
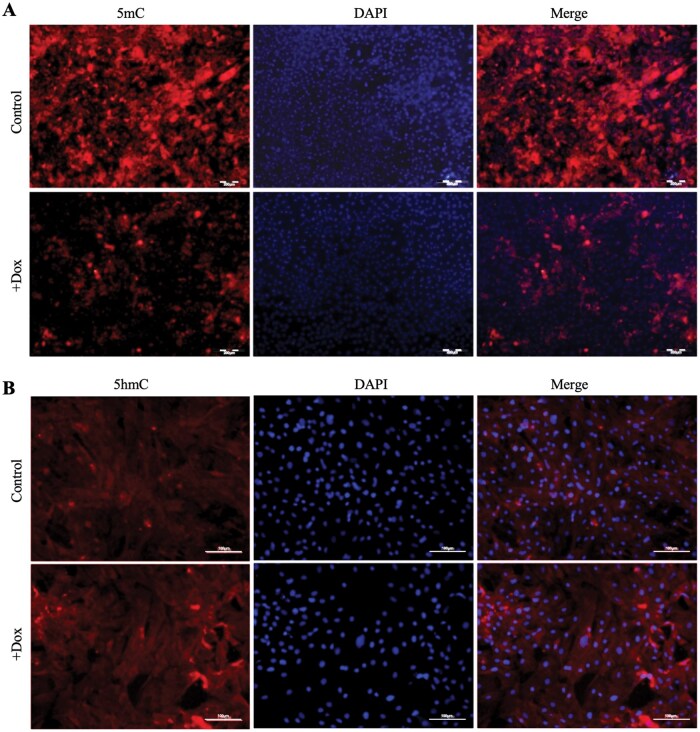
ICC staining analysis of the effect of 20 ng/μL Dox on DNA methylation levels in GTCs. (A) ICC staining analysis of entirety 5mC level in GTCs with 20ng/μL Dox treatment. Scale bar = 200 μm. (B) ICC staining of entirety 5hmC level in GTCs with 20ng/μL Dox treatment. Scale bar = 100 μm.

TET1 catalyzes the oxidation of 5-methylcytosine (5mC) to 5-hydroxymethylcytosine (5hmC), promoting DNA demethylation. To evaluate global methylation changes, ICC was performed using 5mC- and 5hmC-specific antibodies. Dox-induced TET1 overexpression led to a marked reduction in 5mC (Figure 3A) and a significant increase in 5hmC (Figure 3B). Red fluorescence was observed predominantly around nuclei, with reduced unclear 5mC and enhanced 5hmC signals. These findings confirm that TET1 overexpression substantially alters the DNA methylation status of GTCs.

### TET1 knockdown enhances GTC migration

**Figure 4.**
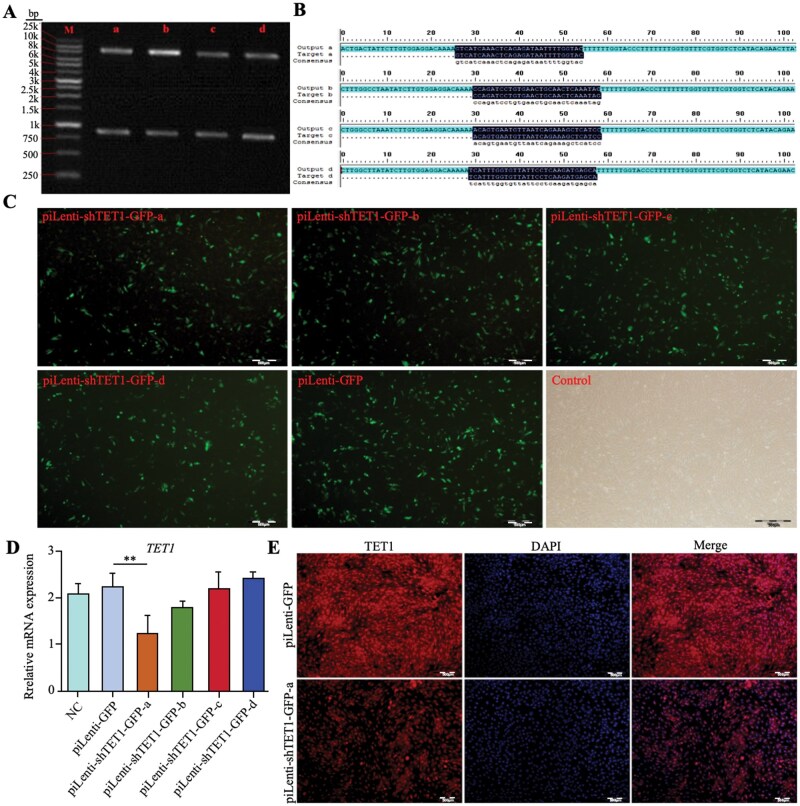
Construction of *TET1* shRNA recombinant plasmid. (A) Kpn I digestion analysis of the TET1 shRNA recombinant plasmid. (B) Sanger sequencing results of the TET1 shRNA recombinant plasmid. (C) Fluorescence detection of transfection efficiency of different TET1 shRNA plasmids in GTCs; scale bar = 500 μm. (D) RT-qPCR analysis of *TET1* mRNA level in GTCs after transfection with different shRNA plasmids; **P < 0.01. (E) ICC staining analysis of TET1 protein expression in GTCs after transfection with piLenti-GFP and piLenti-shTET1-GFP-a; scale bar = 500 μm.

The accuracy of TET1 shRNA constructs was validated by Kpn I digestion, which yielded the expected ∼0.8 kb and 8.2 kb fragments (Figure 4A), and by Sanger sequencing (Figure 4B). GFP-tagged piLenti-shRNA-GFP plasmids were efficiently transfected into GTCs, as indicated by widespread cytoplasmic and nuclear fluorescence (Figure 4C). RT-qPCR identified piLenti-shTET1-GFP-a as the most effective construct, reducing TET1 mRNA to 1.24 relative units compared with controls (*P *< 0.01) (Figure 4D). ICC confirmed decreased TET1 protein expression (Figure 4E). Thus, piLenti-shTET1-GFP-a was selected for furtherstudies.

**Figure 5.**
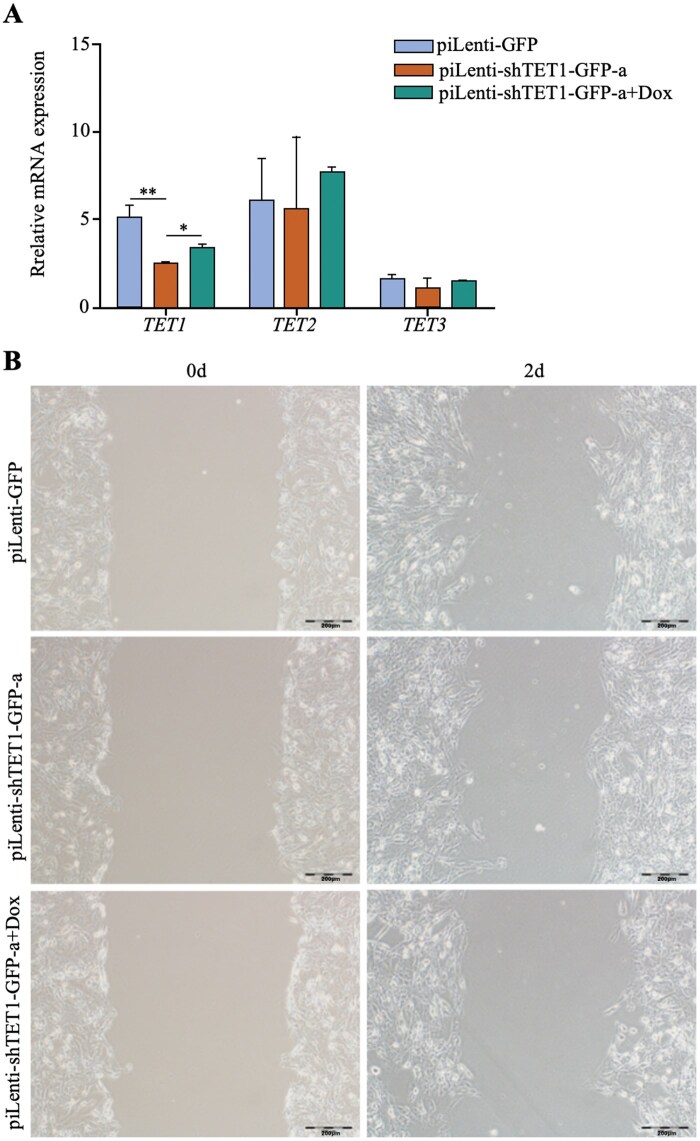
TET1 knockdown enhances the migration capacity of GTCs. (A) RT-qPCR analysis of *TET1*, *TET2*, and *TET3* mRNA levels in GTCs after transfection with piLenti-GFP, piLenti-shTET1-GFP-a, or piLenti-shTET1-GFP-a + Dox; *P < 0.05; **P < 0.01. (B) Wound healing assay analysis of migration ability in GTCs after transfection with piLenti-GFP, piLenti-shTET1-GFP-a, or piLenti-shTET1-GFP-a + Dox; scale bar = 200 μm.

Rescue experiments were conducted by adding 20 ng/µL Dox to TET1-knockdown GTCs (piLenti-shTET1-GFP-a+Dox). Knockdown significantly reduced TET1 mRNA from 4.82 to 2.46 (*P *< 0.01), while Dox partially restored expression to 3.28 (*P *< 0.05) (Figure 5A). TET2 and TET3 remained unchanged (*P *> 0.05) (Figure 5A). Wound-healing assays showed that TET1 knockdown increased migration distance from 127.01 µm to 229.41 µm (80.62%), whereas rescue treatment reduced migration to 43.41 µm, an 81.08% decrease. Compared with controls, rescue restored migration to 146.44% of baseline (Figure 5B). These results demonstrate that TET1 knockdown specifically enhances migration, and the effect is reversible by Dox-induced TET1 overexpression.

### TET1 knockdown increases DNA methylation in GTCs

**Figure 6.**
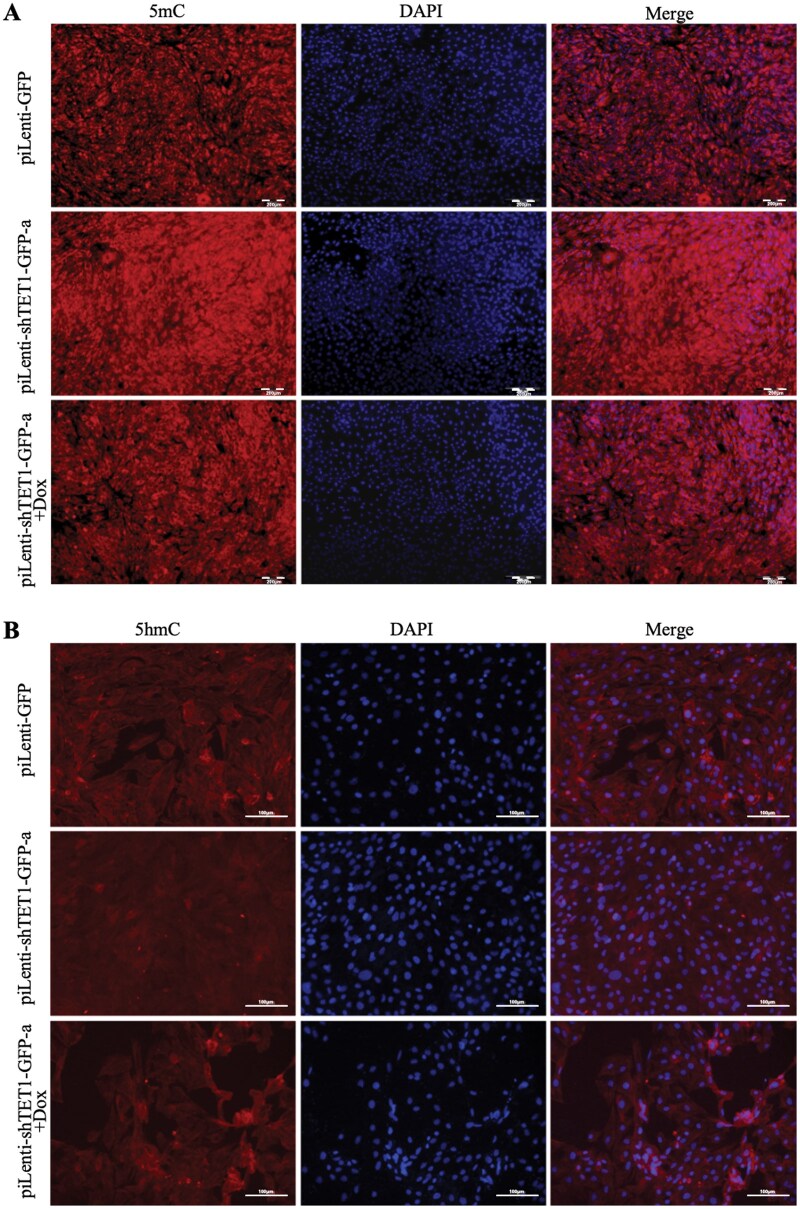
ICC staining analysis of the effect of *TET1* knockdown on DNA methylation levels in GTCs. (A) ICC staining analysis of 5mC levels in GTCs after transfection with piLenti-GFP, piLenti-shTET1-GFP-a, or piLenti-shTET1-GFP-a + Dox; scale bar = 200 μm. (B) ICC staining analysis of 5hmC levels in GTCs after transfection with piLenti-GFP, piLenti-shTET1-GFP-a, or piLenti-shTET1-GFP-a + Dox; scale bar = 100 μm.

To further assess the impact of TET1, ICC analysis was performed for 5mC and 5hmC levels. TET1 knockdown increased global 5mC and decreased 5hmC in both cytoplasm and nuclei compared with controls (Figure 6A, B). In rescue experiments (piLenti-shTET1-GFP-a+Dox), 5mC levels decreased while 5hmC levels increased, partially restoring the methylation pattern. These findings indicate that TET1 knockdown elevates DNA methylation in GTCs, an effect reversible by Dox-mediated TET1 overexpression.

## Discussion

The placenta is a critical component of the maternal–fetal interface, ensuring nutrient and oxygen delivery while removing waste products. Its proper development is essential for establishing and maintaining pregnancy (Ounadjela et al., 2024; Wang et al., 2024a). As a transient organ, it responds dynamically to developmental cues and environmental signals (Aplin et al., 2020; Hemberger et al., 2020). Among placental processes, trophoblast migration is central to mid-gestational development (Wu et al., 2023), yet its regulatory mechanisms in goats remain poorly understood. In this study, we demonstrate that TET1 negatively regulates trophoblast migration by altering DNA methylation in GTCs. Overexpression of TET1 suppressed migration and reduced 5mC levels while increasing 5hmC, whereas knockdown enhanced migration with opposite methylation effects. Rescue experiments further confirmed the specificity of TET1 action. These findings expand our understanding of TET1 in ruminant placental biology and provide a basis for exploring pregnancy-related disorders.

Trophoblast migration and invasion are key steps in placentation and are tightly regulated by transcription factors, signaling pathways, and epigenetic mechanisms (Kohan-Ghadr et al., 2016). During normal pregnancy, trophoblast migration and invasion must be tightly balanced. Excessive invasion can cause placenta accreta spectrum (PAS) (Zang et al., 2024), whereas insufficient migration is linked to preeclampsia (PE) and fetal growth restriction (FGR) (Gong et al., 2024). Our results suggest that TET1 acts as a “molecular brake” on trophoblast migration. This interpretation is consistent with clinical observations of elevated placental TET1 expression in PE patients, where trophoblast invasion is impaired (Gong et al., 2024; Shi et al., 2025). Conversely, reduced TET1 expression has been associated with early pregnancy loss in humans (Wu et al., 2018). As a dioxygenase, TET1 catalyzing the conversion of 5mC to 5hmC, dynamically regulating transcriptional. Our data shown an inverse relationship between TET1 expression and trophoblast migratory capacity, consistent with studies in human trophoblasts where TET1 demethylation of the TGFB1 promoter suppressed invasion (Jiang et al., 2023). Interestingly, these results contrast with cancer biology, where TET1 often promotes migration and invasion (Wang et al., 2024b; Rahim et al., 2025). Nonetheless, multiple studies have demonstrated that TET1 can act as a negative regulator of cell migration. For example, in renal carcinoma, TET1suppresses proliferation, migration, and invasion through apoptosis induction (Fan et al., 2015). Similarly, in basal-like breast cancer, it enhances invasion through POU4F1 activation, promoting cell cycle progression and invasion, whereas TET1 inhibition reduces metastatic potential (Zhang et al., 2024a). In the placental, TET1 has also been reported to promote invasion via HIF1α signaling (Zhu et al., 2017). These seemingly divergent findings suggest that TET1 exerts context-dependent roles in regulating cell migration, highlighting the need for further mechanistic studies to clarify its function in goat placental development.

DNA demethylation is a fundamental epigenetic process regulating gene activity, with the oxidation of 5mC to 5hmC by TET enzymes representing a central step (Vasconcelos et al., 2023). Accumulating evidence has underscored the essential role of TET1 in facilitating active DNA demethylation across diverse biological systems. For instance, Prasasya et al. (2024) demonstrated that TET1 mediates iterative oxidation of imprinting control regions during germline reprogramming, thereby establishing sperm-specific methylation landscapes. Similarly, MacArthur et al. (2024) reported that TET1-dependent demethylation reshapes the epigenetic landscape in neural stem cells, influencing lineage specification and neurogenic potential. In addition, Palczewski et al. (2025) showed that nitric oxide suppresses TET enzymatic activity, resulting in abnormal accumulation of 5mC/5hmC across the genome, further confirming the regulatory flexibility of TET1 in response to cellular metabolic signals. Collectively, in mediating DNA demethylation across a range of physiological contexts. Consistent with these findings, our results demonstrate that TET1 overexpression reduces global 5mC and increases 5hmC in GTCs, whereas TET1 knockdown produces the opposite effect. These data strongly support the conclusion that TET1 acts as a negative regulator of global 5mC and a positive modulator of 5hmC in goat trophoblast cells. By influencing DNA methylation, TET1 may regulate trophoblast migration through gene activation. For instance, TET1 can activate PTEN by promoter CpG demethylation and 5hmC accumulation, thereby suppressing cancer cell invasion (Pei et al., 2016). Such evidence underscores CpG demethylation as a key regulatory switch for transcriptional activation. Beyond single-gene regulation, TET1 may also shape chromatin architecture and pathway balance (Tian et al., 2024). Based on our findings, we propose that TET1-mediated DNA demethylation represent an important epigenetic mechanism controlling trophoblast migration in goat. However, the present work was limited to in vitro experiments and did not address downstream pathways. Future studies should focus on in vivo validation and investigate candidate signaling mechanisms, such as PI3K/AKT and Wnt/β-catenin, to further clarify TET1-mediated regulation of trophoblast behavior.

## Conclusions

In summary, our study demonstrates that TET1 functions as a key negative regulator of trophoblast migration in goats, likely through modulation of DNA methylation dynamics. These findings provide novel mechanistic insights into the epigenetic regulation of ruminant placental development and highlight TET1 as a potential target for improving reproductive efficiency and mitigating pregnancy-associated disorders in livestock.

## Data Availability

All data analyzed during this study are available in the article and/or supporting information, further inquiries can be directed to the corresponding author on reasonable request.
